# Correction: Enhanced charge transport properties of an LFP/C/graphite composite as a cathode material for aqueous rechargeable lithium batteries

**DOI:** 10.1039/d3ra90089d

**Published:** 2023-09-15

**Authors:** Wenyuan Duan, Mubasher, Yanlin Li, Najeeb ur Rehman Lashari, Yuhuan Yang, Cheng Ma, Yuzhen Zhao, Xiaorui Li

**Affiliations:** a Xi'an Key Laboratory of Advanced Photo-electronics Materials and Energy Conversion Device, Xijing University Xi'an 710123 China; b Materials Research Laboratory, Department of Physics, Faculty of Sciences (FOS), International Islamic University (IIU) H-10 Islamabad 44000 Pakistan; c School of Materials Science and Engineering, Xi'an University of Architecture & Technology Xi'an 710055 China liyanlin@xauat.edu.cn; d Institute for Advanced Study, Shenzhen University Shenzhen 518060 China najeeblashari@xpu.edu.cn; e Department of Chemistry and Physics, Jackson State University USA; f School of Environmental and Chemical Engineering, Jiangsu University of Science and Technology Zhenjiang 212114 P. R. China

## Abstract

Correction for ‘Enhanced charge transport properties of an LFP/C/graphite composite as a cathode material for aqueous rechargeable lithium batteries’ by Wenyuan Duan *et al.*, *RSC Adv.*, 2023, **13**, 25327–25333, https://doi.org/10.1039/D3RA04143C.

The authors regret that the name of one of the authors, Mubasher, was given incorrectly in the original manuscript. The corrected list of authors for this paper is as shown above.

The Royal Society of Chemistry apologises for these errors and any consequent inconvenience to authors and readers.

## Supplementary Material

